# What factors explain extreme sport participation? A systematic review

**DOI:** 10.3389/fspor.2024.1403499

**Published:** 2024-07-16

**Authors:** Odette Hornby, Gareth Roderique-Davies, Robert Heirene, Elin Thorkildsen, Sophie Bradbury, Iwan Rowlands, Egan Goodison, Jodie Gill, David Shearer

**Affiliations:** ^1^ Faculty of Life Sciences and Education, University of South Wales, Pontypridd, United Kingdom; ^2^ School of Psychology, University of Sydney, Sydney, NSW, Australia

**Keywords:** extreme sport, motivation, participation, personality, risk

## Abstract

**Objective:**

Extreme sport participation is growing, yet it is still not clear exactly what motivates individuals to participate in sports where accidents can lead to serious injury or death. The purpose of this systematic review was to review and assess current research and identify the factors that explain engagement in extreme sport participation.

**Methods:**

A systematic review of PsycInfo, ProQuest, PsychArticles, SportDiscus and Google Scholar was performed according to PRISMA guidelines. Eligibility criteria were defined to identify studies exploring the factors that explain or are associated with taking part in extreme sports. Articles published in English in peer-reviewed journals were retrieved.

**Results:**

A total of 35 studies met the eligibility criteria. The sample comprised 17 qualitative studies, 12 quantitative studies, 5 case studies and 1 mixed method study. Findings were categorised into five key themes; “*existential and external*” (external reasons for participation, e.g., being in nature), “*personality*” (i.e., stable traits that predicted participation e.g., sensation seeking), “*motivation characteristics*” (i.e., one's capability and confidence whilst participating, e.g., self-determination theory), “*managing risk*” (i.e., explanation centred around the desire to take risks e.g., experiential vs. analytical) and “*analogies with addiction and withdrawal*” (i.e., the behavioural response experienced whilst abstaining from/unable to take part in the sport e.g., craving).

**Conclusion:**

There are multiple reasons why individuals participate in extreme sports despite their inherent danger. This review highlights how individuals differing perceptions of risk can impact motivations and therefore the complexity in this area. Potential links between themes and suggestions for future research are also discussed.

**Systematic Review Registration:**

https://osf.io/mvk2j.

## Introduction

Participation in extreme sports has grown exponentially since 2000 and due to increased media coverage (tv, films, documentaries, news reports), has continued to grow (1–3). The notion that extreme sports are exclusively for the young is evolving, with participation rates increasing across different generations (4). For instance, baby boomers are actively participating in extreme sports (2) while Gen Z are drawn to extreme sports due to their increasing popularity. Since the COVID-19 pandemic, Extreme International (a large-scale media brand that creates a global community within extreme sports) has noted substantial increases in participation which they have attributed to an increased willingness to travel and a larger desire to step out of comfort zones (5). The worldwide extreme sport market is estimated to bring in over $200 billion per year with around 490 million participants globally, showing the scale of participation (5). The most common extreme sports include, but are not limited to, rock climbing, cliff diving, mountain biking, BASE jumping, wing suit flying and big wave surfing (6).

There is currently a lack of consensus over the exact definition of what an extreme sport is (7) and the terms that are used, often leading to researchers using these terms interchangeably based on their own definition (e.g., adventure sports, high risk, action, alternative, lifestyle sports). The initial definitions used by Brymer (8) and Breivik et al. (9), defined extreme sport as one in which a mismanaged mistake or accident would result in serious injury or death and was inherent to the activity. However, newer definitions suggest extreme sport is “a (predominantly) competitive (comparison or self-evaluative) activity within which the participant is subjected to natural or unusual physical demands.” Moreover, an unsuccessful outcome is “likely to result in the injury or fatality of the participant, in contrast to non-extreme sport” [(10), p. 138]. Another definition by Boudreau et al., (11) defined extreme sport in their review as “a self-initiated nature-based physical activity that generates heightened bodily sensations […] and requires skill development to manage unique perceived and objective risks” (p. 2). Despite these newer definitions, exactly what constitutes extreme sport is still not clear (12) and challenges remain when defining extreme sport, often leading to researchers creating their own criteria (13). However, the element of objective personal risk is consistent across extreme sports.

Researchers have examined several factors that may explain why individuals participate in extreme sports despite the risks associated with them; these include immersion in nature, sensation seeking, alexithymia, anhedonia, withdrawal, craving, rush and flow (14–18). However, it remains unclear which constructs most consistently and strongly explain extreme sport participation across studies and sports. Further, while studies have explored the role of factors like the environment, personality traits, states (e.g., rush) and neurobiology *separately* (19, 20), there has been no integrated discussion of these to date.

The purpose of this systematic review (SR) was to (1) review and assess current research and identify the factors that explain engagement in extreme sport participation, and (2) provide an integrated discussion of the various factors across disconnected domains of extreme sport research (e.g., personality traits, motivation, environment). The review provides important insights into our understanding of human motives and behaviour which may extend beyond the realm of extreme sports. By improving our understanding of why people engage in extreme sports, it may offer a lens through which we can understand participation in other dangerous or high-risk domains and vocations (e.g., deep-sea diving, military service).

## Methods

The methods used in this systematic review (SR) were registered *a priori* on the Open Science Framework. The protocol can be accessed here: https://osf.io/mvk2j.

## Eligibility criteria

Eligible studies focused on exploring the factors that explain or are associated with taking part in extreme sports, including but not limited to qualitative studies of motivations to participate, cross-sectional studies comparing the characteristics (e.g., personality traits, demographic characteristics) of extreme sports athletes with other sports, and (neuro) biological investigations. Based on the definition stated in the introduction, any sport that puts individuals at risk of serious injury or death in a natural environment were included.

Inclusion criteria were as follows:

- Must focus on exploring the factors that explain why people take part in extreme sports.
- Studies must be published in English.
- Participants must be 18 years or older.
- Study participants must have taken part in extreme sport for at least 6 months.
- No restrictions were placed on study designs or year of publication.
- Papers must be peer-reviewed.

Exclusion criteria were as follows:

- Heli-skiing as it is now banned across Europe.
- All contact sports (i.e., Rugby) were excluded as they involve competing directly against someone rather than within nature.
- Any sport involving motorised technological innovation (i.e., F1 or motocross) as technology may determine the risk involved.
- No reviews were included; however, they were used to find relevant sources.

## Information sources

The final search was conducted on January 4th, 2024. The following databases were searched to obtain relevant articles: PsycInfo, ProQuest, PsychArticles, SportDiscus and Google Scholar (as a secondary data base). Reference lists of all included articles were searched for suitable studies. A list of included studies was circulated to all authors of this review to ensure all relevant literature was identified. To identify any unpublished or on-going studies, leading researchers in the field were contacted.

## Outcome

### Main outcome

To understand the biological, psychological, social, and environmental factors that contribute to individuals’ participation in extreme sport.

## Search strategy

The search strategy was developed by the lead author (OH). OH consulted a thesaurus and engaged in discussions with the research team and university librarian to identify all possible search terms. Search terms were developed for: (1) the different types of extreme sports (e.g., dangerous sports, adventure sports etc.) and (2) factors relating to/explaining participation (e.g., rush, sensation seeking etc.). The main databases were searched using a predefined set of terms: (“extreme sport” OR “high risk sport” OR “adventure sports” OR “adrenaline sports” OR “risky sports” OR “dangerous sports” OR “BASE jumping” OR skydiving OR “rock climbing” OR mountaineer* OR “big wave surf*” OR “mountain bik*” OR “free soloing” OR “bungee jumping” OR “cliff diving”) AND (motivat*on OR incentive OR purpose OR reason OR factors OR explanations OR flow OR “sensation seeking” OR rush OR withdrawal OR craving OR anhedonia OR alexithymia OR wellbeing OR “outdoor exposure”).

## Selection process

**Figure 1:**
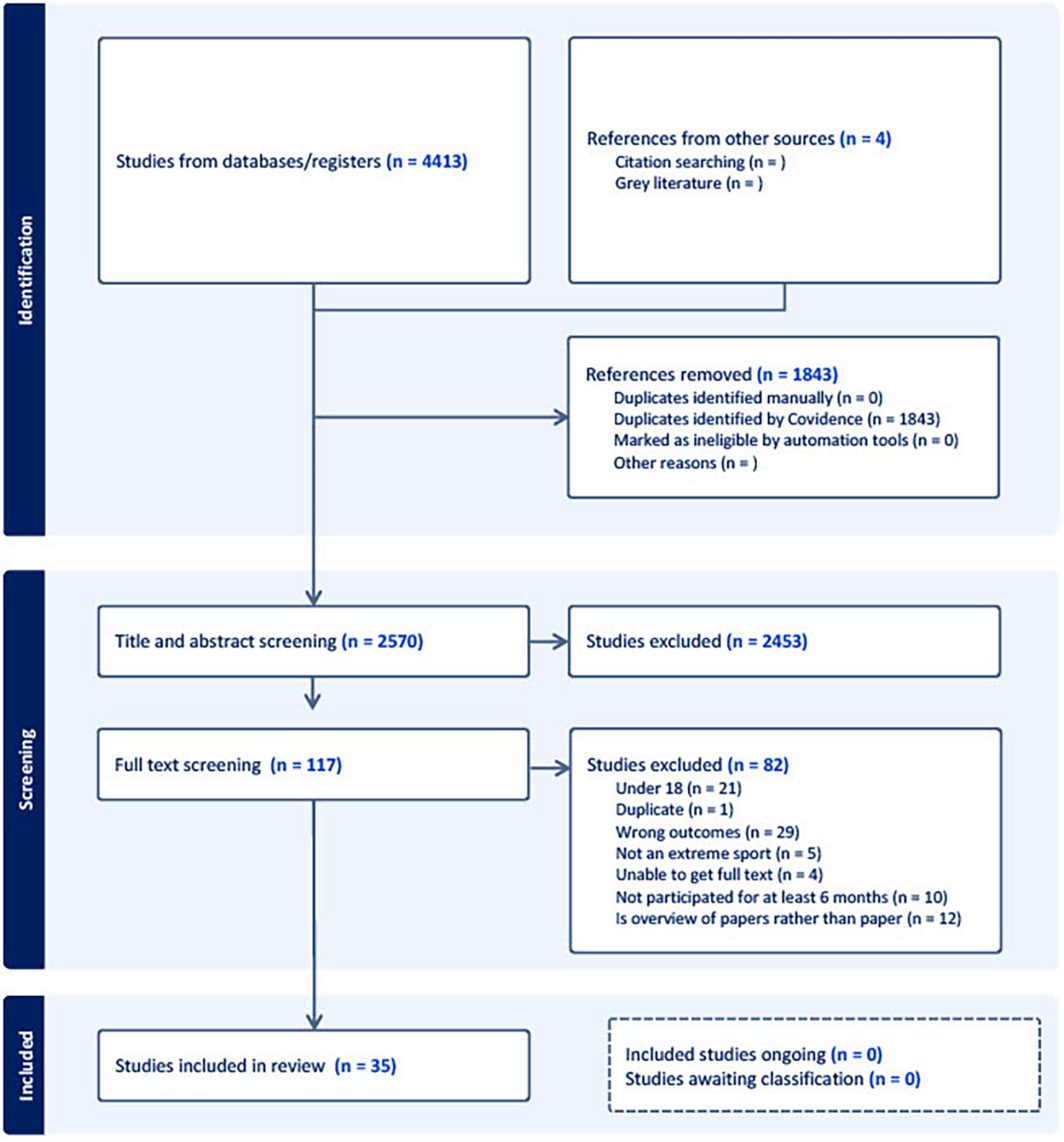
PRISMA flow diagram.

Studies were uploaded to Covidence, an online software program designed specifically for collating and screening studies for systematic reviews. The lead reviewer (OH) screened titles and abstracts identified by the searches for potentially relevant studies. A second and third screener (SB & IR) were recruited to ensure consistency and agreement on chosen studies. Of those deemed potentially relevant, reviewers independently assessed the full text against the inclusion criteria. Any disagreements were solved through discussion and, if required, a fourth reviewer was consulted. Duplicates were identified and excluded through Covidence and all excluded studies and reasons for their exclusion are detailed in the flow chart (Figure 1) (21).

## Data collection process

Data extraction was conducted by three reviewers independently. Any disagreements were resolved by consensus or by involving a fourth reviewer. Each reviewer independently (OH, EG, JG) read and extracted data and inputted this directly onto Covidence. Once all data was extracted, all three reviewers double checked the data was correct by comparing extracts and ensuring consensus. The following was extracted from included studies:

• Title, author, publication year and journal title
• Sample size, mean age, gender, and sport(s)
• Study eligibility, article availability and language
• Key finding: factor for participating in extreme sport
• Research gap identified—what questions did the research leave us with

Results are presented in Supplementary Table S1.

## Study risk of bias assessment (quality assessment)

Two review authors (OH, ET) independently assessed the risk of bias and quality of each individual study. Using an adapted version of a study assessment tool developed by the National Institute of Health [https://www.nhlbi.nih.gov/health-topics/study-quality-assessment-tools (21)], the following questions were created to ensure limited bias and quality. The questions were adapted to include extreme sport specific questions. A quality assessment checklist was therefore developed with the following questions:

1. Was the research question or objective clearly stated?
2. Was the study population (sample, mean age) clearly specified and defined?
3. Was the extreme sport(s) clearly outlined? (adapted question)
4. Were the factors for participating in extreme sport clearly identified? (adapted question)

Results are presented in Supplementary Table S2.

The same two reviewers discussed any disagreements, and a third reviewer was consulted to resolve any differences. Each question required a response of “Yes,” “Unclear” or “No.” If all questions were answered “Yes” then the paper completed all quality assessment requirements and was therefore sufficient to be used in the review. For all included studies we also assessed them on four measures of transparency and quality:

1. Was the study pre-registered?
2. Is the study data openly shared and accessible?
3. Are the study materials (e.g., experimental stimuli, study-specific questionnaires/interview guides) openly shared and accessible?
4. Does statcheck (where applicable) identify any statistical reporting errors?^1^

Results are presented in Supplementary Table S3.

## Data synthesis

A formal descriptive and narrative synthesis of the studies was performed based on the outcomes of the selected papers. Thematic synthesis was used to analyse the results, a method described in detail by Thomas and Harden (24). Initially studies were read, and the main characteristics were identified, along with possible “descriptive themes” and results. When all studies had been examined and discussed more than once, similarities, differences and relationships between them were considered. This then allowed us (OH, DS) to create “analytical themes.” Based on guidelines set out by the Cochrane Consumers and Communication Review Group (25) the review also followed three stages:

1. Develop a preliminary synthesis of the findings of the included studies.
2. Explore the relationship in the data within and between studies.
3. Assess the robustness of the synthesis.

## Results

**Figure 2:**
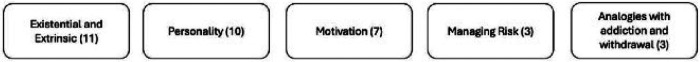
Allocation of papers to theme.

The online electronic searches identified 4,413 results. After removal of duplicates (1,843) and title and abstract screening, 117 articles were selected for full text screening, with 35 subsequently identified as relevant to this systematic review. A PRISMA flow diagram showing an overview of the identification and screening process can be seen in Figure 1 along with the reasons for study exclusion (21). Due to the range of outcomes reported, studies were grouped into five overarching categories (Figure 2). Specifically, eleven to “*existential and external*” (i.e., research that focuses on external reasons for participation e.g., being in nature), ten studies were assigned to “*personality*” (i.e., studies that focussed on stable traits that predicted participation), seven to “*motivation characteristics” (*i.e., one's capability and confidence whilst participating*),* three to “*managing risk*”(i.e., explanation centred around the desire to take and manage risks), and three to “*analogies with addiction & withdrawal*” (i.e., the behavioural response experienced whilst abstaining from the sport). Some of the studies had multiple themes that crossover but have been placed where they address the most/important points. Themes are presented in the order of those with the most studies first, progressing to those with the least.

## Participant characteristics

### Demographics

Study sample size varied from one (26–28) to 7,109 participants (29). Although many studies did not disclose mean ages (*n* = 12), in those that did (*n* = 22), mean age of participants ranged from 19.68 years (29) to 36 years (30). Seven studies used male-only participants (18, 28, 31–35), three studies used female-only participants (26, 27, 36) and the remaining studies used mixed-gender or did not disclose participants' gender. Inclusion of transgender or gender fluid participants was not reported by any study.

### Recruitment

Sixteen studies recruited participants using purposive sampling technique [e.g., (18, 37, 38)], three using convenience sampling (27, 39), two using snowballing (40, 41), one using a combination of convenience and snowballing (42) and twelve studies did not disclose sampling methods despite having details of the inclusion criteria in their methods [e.g., (43–45)].

## Study design

Seventeen articles used qualitative methods [e.g., (44, 46, 47)], twelve used quantitative methods [e.g., (31, 45, 48)], five were case studies [e.g., (26, 27)], and one used a mixed method design (49). The most common data collection methods were interviews [23 studies e.g., (32, 50, 51)] and psychometrics or surveys [12 studies e.g., (29, 43, 52)].

## Themes

### Existential and extrinsic

The existential and extrinsic theme consisted of eleven papers and was developed to encompass factors within the included studies that related to extreme sports that are extrinsically oriented. “Existential” in this context refers to the existence of individuals as free and responsible for determining their own development (53), as it directly relates to human existence. “Extrinsic” refers to the type of motivation that involves striving towards an external goal or being driven by external factors (54). Sub themes such as engaging with nature [e.g., (30, 37, 47, 50, 55)], freedom [e.g., (2, 34, 47, 51)] social interactions [e.g., (30, 37)], and challenge (34, 51, 55, 56) were encompassed within this theme.

The sub-theme of nature was seen as a key motivator for individuals to engage in extreme sports (30, 37, 50), as individuals felt they could explore and appreciate new and remote spaces, giving them a sense of freedom from everyday life (2, 51). When immersed in nature, extreme sports athletes reported a sense of tranquillity and transformation of time when engaging in their sport (41). This was reported to increase their feeling of being present in the moment, which is analogous to the concept of mindfulness (56). Focusing on the immediate task in this way was said to distract them or allow them to shift attention away from unpleasant and/or stressful feelings (56). This distraction in nature could also be seen as a sense of freedom. For example, Brymer and Schweitzer (2) found six key elements of freedom, which included; freedom from constraints; freedom as movement; freedom as letting go of the need for control; freedom as the release of fear; freedom as choice and personal responsibility; and freedom as being at one. The social interaction was seen to motivate individuals as they could enjoy challenges with friends and meet new people, allowing them to feel a sense of “belonging” (37). The subtheme of challenge was discussed by studies in the review as a key motivation for participation [e.g., (34, 56)]. For example, Frühauf et al. (51), found challenge motivated athletes in many ways (encountering new places, exploring personal limits, experiencing skill, overcoming the challenges of environmental conditions). Pushing limits was seen to be both a mental and physical demand (56).

### Personality

Personality included ten papers and was discussed in relation to participation/motivation for extreme sports, either as individuals having a sensation seeking (SS) trait or the stable trait alexithymia leading to the use of extreme sports as a means of emotion self-regulation. Sensation seeking (SS) is a trait typified by the propensity of individuals to seek high-risk activity that provides thrills and excitement (57). Alexithymia is typified by individuals who have difficulty identifying and describing their emotions (17), which can lead many individuals to use extreme sport as a means of emotion self-regulation (17, 42).

### Motivation

The theme motivation includes seven papers and aims to explain individuals' motives for participation in extreme sport. Self-determination theory's (SDT) elements of competence, autonomy and relatedness all connected to athletes' motivations. Athletes have reported to experience all three elements of SDT when engaging in challenging situations, leading them to seek out these experiences more (58). Extreme sport athletes reported feeling a sense of autonomy over choices regarding their activity, increasing their perception of control over their experience. This is said to give athletes the feeling of complete freedom to decide when, where and how to engage in these activities (39). Extreme sport athletes reported that motivation linked to goal achievement in the form of winning competitions (59), potentially contributing to the fulfilment of competence. The element of relatedness can be fulfilled through social support and the sense of belonging with like-minded individuals. In the studies reviewed this was said to create strong feelings of relatedness due to the camaraderie experienced (60).

Constructs such as self-efficacy refer to beliefs in one's capabilities to organise and execute a particular skill or activity (61). As confidence and self-efficacy increase, research suggests individuals may take more risk, challenge themselves to harder goals, expand their effort and persist in the face of adversity as they believe they are better equipped to cope with the situation (33, 62). Extreme sports athletes reported a sense of achievement when participating, particularly from setting goals or winning competitions (33, 46). This could be attributed to achievement goal theory which suggests that an individual's vision can be converted into goals, initiatives and activities that help direct behaviours and actions in specific ways (63).

### Managing risk

In the literature reviewed, the concept of risk was examined with regards to individuals’ motives for participating in extreme sports (three papers). Risk can be split into two main mechanisms: analytic and experiential. Analytic risk refers to conscious, rational, and logical decisions that require information to justify a conclusion. Whereas experiential is unconscious, automatic, and requires no justification or prior knowledge. Participating in extreme sports is thought to put an individual at both physical {injury [e.g., (29)] or death} and psychological (high-stress, competitiveness, and perfectionism) risk (64). However, some participants appear attracted to risk situations as desirable rather than something that should be minimised (65).

### Analogies with addiction and withdrawal

The analogies with addiction and withdrawal theme encompassed three papers that explain an alternative motivation for participating in extreme sport. Withdrawal states such as anhedonia, craving and negative affect reported by extreme sport participants [e.g., (18)] bare similarities to the emotional experience of people with addictions (i.e., substance and behavioural). Of relevance, behavioural addictions are classified by excessive engagement, mood modification, withdrawal, conflict, and relapse (66). Some of the literature [e.g., (18)] indicated that individuals continually engage in extreme sports as when they do not participate, they experience mood disturbance (e.g., anhedonia), which may lead to a powerful desire to continually engage (e.g., craving) [e.g., (28, 40)]. When individuals stop or break from participating (e.g., rest days), they may experience physical and psychological responses (i.e., withdrawal).

## Discussion

The purpose of this review was to examine the factors that seek to explain engagement in extreme sports. The key domains of interest were existential and extrinsic, personality, motivation, managing risk, and analogies with addiction and withdrawal. Each domain will be discussed separately for clarity and ease, followed by suggestions for how an integrated view can or might be developed.

### Existential and extrinsic

Several reviewed studies highlighted extrinsic motivators such as nature [e.g., (27, 30, 47, 50, 51, 55)], freedom [e.g., (2, 47, 51)] social interactions [e.g., (27, 30, 37)] and challenge (51, 55, 56). Studies on these factors were predominately interview based [e.g., (2, 30, 51)], allowing rich insights into how they related to participation. However, these extrinsic motivators can be deemed subjective, making it difficult to compare across individual data. It is therefore unclear if individuals within extreme sport perceive the outdoor element of nature the motivation for their participation, or whether any outdoor sport would give the same reward. Given athletes highlighted that extreme sport gives them a feeling of freedom from constraints [e.g., (2)], it is also unclear if the feeling of freedom is due to being immersed in the outdoors or because of their sport.

In relation to social connection, extreme sports athletes appear to be motivated by the camaraderie with like-minded others, giving a sense of belonging [e.g., (27)]. This bares similarities with research on those in the military, another high-risk domain (67). Individuals in military environments often suggest they feel part of a “family” due to their shared experience with like-minded individuals in the same career, often described as “brothers or sisters” (68). However, research has also suggested low risk sports can also lead to camaraderie leading to feeling part of a team collective (69). Thus, camaraderie may not be unique to extreme sport participation and individuals could experience this benefit from low-risk sports.

### Personality

Personality is commonly explored in studies attempting to understand the motivation to participate in extreme sport. The studies reviewed here indicate that personality motivates individuals to participate in extreme sport due to the sensations of thrill (45) and the ability to “feel” and therefore describe feelings (49). Two core themes relating to personality were identified: (1) sensation seeking and (2) alexithymia and emotion self-regulation.

Some studies found individuals are motivated to take part in extreme sports for the thrills and excitement experienced whilst participating [e.g., (45, 48, 52)]. For example, Slanger and Rudestam (45) explored a range of sports and found those in extreme sport scored significantly higher in the thrill and adventure seeking aspect of the sensation seeking scale than low risk sport participants. Furthermore, some authors have suggested that extreme sport athletes may become desensitised to “thrill” with continual exposure, which might lead them to continually seek out either new or more extreme thrills (70). For example, Fruhaüf et al. (30), found that individuals continually expanded and relocated their perceived limits to seek new thrills. This suggests that when extreme sport athletes participate for reasons related to sensation seeking, their participation becomes self-perpetuating.

Most of the studies on sensation seeking reviewed in this SR used Zuckerman's sensation seeking scale as a measurement tool (31, 45, 48, 52). This scale was initially developed to look at individuals' propensity to engage in activities rather than motivation to participate (42). This makes it difficult to conclude that participants in these studies were motivated by the sensations they feel [e.g., (31, 45, 48, 52)]. For example, Cronin (52) found mountain climbers scored higher on experience seeking and thrill and adventure seeking than the control group, however, it is not clear how (if at all) sensation seeking motivates extreme sports athletes.

There are several additional issues with Zuckerman's scale. First, there are concerns regarding the outdated nature of the scale. The vocabulary used such as “swingers” and “queer” highlights this problem, as these words may be seen as offensive and stigmatising (71). This could result in individuals not feeling comfortable to answer certain questions or the questionnaire as a whole. Second, the use of forced response may leave many individuals feeling they could have responded to either both options or neither (72), this increases the likelihood of misrepresentation in individuals' responses. Lastly, when understanding extreme sport athletes motivations, the only relevant aspect of the scale is the *thrill and adventure* seeking element. For example, the disinhibition element of sensation seeking focuses solely on participating in drug use, alcohol and sex which is not relevant to extreme sport motivation. However, if an extreme sport version of the sensation seeking scale for disinhibition was developed this could look at disinhibition in relation to disregard for risk.

Despite these weaknesses, the scale has been continually developed across the years to reduce these issues (73). Whereas it was initially focused on examining the need for novelty and complexity, this has changed to a focus on novelty and intensity, as Zuckerman considered this a defining characteristic of sensation seeking (73). The intensity aspect is important as it determines the vigour of attention and therefore the duration and length of time a sensation persists. However, despite the changes to the sensation seeking scale over time, the role of sensation seeking in explaining extreme sports participation remains unclear. For example, Jack and Ronan (74) found swimmers scored higher on measures of sensation seeking than some high-risk sports participants (hang gliding & motorsports). Anomalies like this suggests that the sensation seeking scale may be limited in its utility to explain extreme sport participation. Therefore currently, sensation seeking may not be a useful differentiation between extreme sports athletes and other groups. More recently, the Sensation Seeking, Emotion Regulation and Agency Scale [SEAS (43)] scale was developed to measure experiences during, after and between participation in extreme sport (43). As contrast to Zuckerman's sensation seeking scale, the SEAS aims to measure and understand motives for seeking emotion regulation and agency through extreme sport participation. It is the first scale that highlights the distinctions in individual differences, behaviour and extreme sports (e.g., skydiving vs. mountaineering). Given the recency of the SEAS scale, it uses appropriate language in comparison to Zuckerman's sensation seeking scale.

Alexithymia is typified by individuals who have difficulty identifying and describing their emotions (17) and is related to emotion self-regulation. Emotion self-regulation refers to one's ability to initiate and maintain the type, intensity and duration of the emotions they feel (75). Three reviewed studies suggest that extreme sport athletes may have difficulty expressing emotions (alexithymia) [e.g., (17, 36)] and their sport provides an opportunity for them to experience greater emotion regulation [e.g., (42, 49, 76)]. Woodman et al. (49) used the Toronto Alexithymia Scale (TAS-20) to assess three aspects of the alexithymia construct [i.e., (1) difficulty identifying feelings (2) difficulty describing feelings (3) externally orientated thoughts]. Woodman et al. (49) suggested that transatlantic rowers exhibited significantly higher scores in describing emotional difficulties compared to normative values derived from an English-speaking adult population. Engaging in rowing appeared to facilitate emotional identification and expression for participants. However, individuals with high levels of alexithymia might struggle to accurately assess their emotional awareness (77). Thus, rather ironically, research relying on the TAS-20 scale to indicate alexithymia may not fully capture an individual's emotional perception. To overcome this issue, an interview method [Toronto Structured Interview for Alexithymia (TSIA)] has been developed, although this exact method (TSIA) was not used in any of the reviewed studies (78).

Studies examining the role of emotion regulation in extreme sport participation used the SEAS [e.g., (43)]. Results across studies indicate that extreme sport athletes experience difficulties in emotion regulation, although this can differ across sports (i.e., mountaineers vs. skydivers. Freeriders vs. slope skiers) [e.g., (38, 43)]. For example, Frühauf et al. (38) found freeriders experienced higher emotion regulation than slope skiers during and after participation in their sport but not between participation.

### Motivation

Reviewed studies suggested that the more self-efficacy individuals have for what they can do in their sport the more risk they were willing to take (62). In general, as confidence and self-efficacy increase, individuals are thought to take more risk, challenge themselves to harder goals, expand their effort and persist in the face of adversity as they believe they can cope with the situation (33, 62). In the context of extreme sport, Jones et al. (32) found an individual's perceived ability was an important factor in decision making when embarking on the most difficult winter climbing routes. Further, Wiersma (35) found when surfers' confidence increased, so too did their desire to strive for bigger waves. They also found that as experience increased, competence and confidence also increased, which led athletes to believe they were taking less risk. This interplay between experience, confidence and risk perception is similar to the definition provided by Boudreau et al. (11).

In the reviewed studies, there was also a potential link to achievement goal theory suggesting that individual's visions are converted to goals. Burke et al. (46) found setting goals allowed climbers to feel as prepared as possible going into a specific task and gave a sense of achievement at the end. However, not all individuals felt this, as they believed that having too many outcome-based goals reduced the element of fun. Mackenzie (59) found that participants in varied extreme sports (kayaking, downhill mountain biking, mountaineering, BASE jumping, skydiving and hang gliding) predominately found goal setting useful for self-focus and competitive states but also appreciated goal achievement in the sense of winning competitions and medals. This suggests that extreme sport athletes strive for outcome-based goals, similar to more traditional sports where athletes typically set goals to enhance performance (79, 80).

### Managing risk

Extreme sport athletes tend to exhibit a diminished concern for the potential risk and consequences involved, most of this is due to how they manage the risks they take. Such attitudes towards risk are often attributed to athletes ability to mitigate and attenuate the perceived risk through rigorous training, preparation, and control [e.g., (44)]. For example, Brymer et al. (44) found that “outsiders” (low risk and non-sporting individuals) viewed extreme sport as risky but those that participate in them viewed it as a means for emotional clarity and aim to mitigate the risk involved. Despite this Weishaar et al. (29), found that risk seeking and lack of perseverance were the two strongest predictors of extreme sports injury, highlighting that extreme sport does hold some level of risk. The concept of risk is subjective (81) and so it is difficult to compare risk perceptions between individuals, particularly when using interviews as a data collection tool—as was frequently the case in the reviewed studies. As well as being subjective, understanding risk is relative. For example, some individuals may find going for a walk in the mountains as risky, whereas another person in the same sex and age bracket may find this exciting. This is relative to an individual's perception and skill level. In a case study by Kerr (26) the athlete made the decision to withdraw from skydiving following a “freak accident” and death of a friend. The uncontrollable death and therefore risk became too high for the athlete to continue participating. Similarly, a number of high level of extreme sport athletes have quit due to risk perceptions and changes in circumstances. For example, Tim Emmett publicly discussed quitting base jumping following the death of a friend and the birth of his child (82).

In recent years, the measurement of risk has developed with the introduction of the risk-taking inventory scale, which was devised to measure attitude and propensity to take risk (83). However, despite these developments, no studies within this systematic review used this new scale to determine risk, making it difficult to understand whether included studies represent current thinking in the literature or support the inclusion of attitude and propensity.

### Analogies with addiction and withdrawal

A small number of studies found evidence for withdrawal states such as anhedonia, craving [e.g., (40)] and negative affect being experienced by extreme sport participants [e.g., (18)]. Heirene et al. (18) found that climbers experienced more frequent and intense anhedonia, craving and negative affect where “nothing compared to climbing.” These symptoms bare similarities to those individuals with behavioural addictions (84). However, it is important to note that there were limited papers in this systematic review that discussed addiction in the context of extreme sport. Popular theories and perspectives in the substance and behavioural addiction literature stipulate that several additional criteria would be required for individuals to be considered as having an “addiction”– namely, impaired control over the behaviour and negative personal, social, or occupational consequences associated with it (85, 86). Several studies in this review examined specific reasons given for participation [e.g., (40)], yet only one (18) explicitly connected it to addiction. However, it was common that symptoms often linked to addiction, such as craving (a desire to experience it again), were prevalent in participants [e.g., (28)]. Without an explicit connection, it becomes challenging to discern whether withdrawal states result from an “addiction” or a deep passion for their sport.

Considering the additional criteria for a behaviour to be viewed as an addiction, a potentially different viewpoint not explored in the literature to date is “passion”. Passion refers to the engagement in an activity an individual finds important and therefore invests time and energy into it (87). Passion can be split into harmonious and obsessive passion. Harmonious passion refers to an individual flexibly and autonomously engaging in a sport. Whereas obsessive passion is when an individual rigidly participates in a controlled way (88). This leads to individuals attaching greater importance on the sport, potentially using it to escape problems and emotions. This in turn makes it difficult to stop the activity, making it hard to conclude if individuals are experiencing withdrawal symptoms similar to those with addiction, or if they are experiencing obsessive passion. However, it is reasonable that obsessive passion could lead to symptoms of withdrawal if an athlete is forced to stop their sport (e.g., injury).

## Integrating themes for future research

Through examining the research in this systematic review, it is clear there are several ways in which the five themes can and may interrelate, which may inform future research direction. Here, we present potential opportunities for researchers to help develop a more integrated understanding of participation in extreme sport. However, this list is certainly not exhaustive. First, evidence suggests there is a potential relationship between personality and risk taking. For example, risk taking is associated with extroverted individuals, as they are willing to put themselves in more dangerous positions (89). Therefore, it could be suggested that having a more extroverted personality may predispose individuals to participate in extreme sport and increase the propensity for risk taking. Theory would also suggest that individuals who have high disinhibition and sensation seeking are more likely to take risks. Extreme sport athletes typically tend to exhibit a diminished concern for the potential risk and consequences involved in their sport [e.g., (55)], also known as disinhibition (90). Disinhibition could manifest as a reduced concern for personal safety behaviours (90). This may lead individuals to push their physical limits without fully considering the potential dangers involved. These individuals may also have higher levels of self-belief and therefore self-efficacy, which Bandura (91) suggests reduces stress reactions. This could lead these individuals striving for more risk as they feel more competent to do so.

Second, there is good reason to suggest a link between alexithymia, anhedonia and withdrawal. For example, those that struggle to express their emotions (alexithymia) may strive to participate in extreme sport to seek sources of intense stimulation to increase arousal rather than the under arousal they experience in day-to day life (anhedonia) (92). This may therefore lead individuals exposed to a higher risk of experiencing anhedonia and alexithymia when not participating, resulting in symptoms of withdrawal and craving that motivates further participation in extreme sport for the coping strategy it brings.

Lastly, theory may lead us to believe that those with a need for connection and nature will experience withdrawal-like states. The link between anhedonia, withdrawal and connection could suggest that individuals feel a heightened sense of withdrawal due to not only withdrawing from the sport but also from withdrawing from the community and nature element. Examining the root of individuals' motivation to participate involves discerning whether they are drawn to the sport primarily for the activity itself or if their connection is more rooted in sharing similar personality traits with others in the environment.

## Limitations

This was (to our knowledge) the first systematic review that has attempted to understand participant motivations in extreme sport. The review process was transparent, systematic, and included an extensive list of articles from a variety of different extreme sports. However, the review may be limited by the small sample sizes in several of the studies and therefore the generalisability of findings beyond the samples (26–28). For example, Kerr (26) used a single case study approach with a female athlete about her motivational experiences during skydiving. Many of the studies used in the review were also predominately male based [e.g., (18, 31, 32)]. This makes it difficult to generalise the findings to females who may have different motives, which is especially important due to the increased participation of women in extreme sport (93).

Second, there was heterogeneity in defining extreme sport. As there is no definitive list of extreme sports, or an agreed definition, it becomes difficult to decipher what sports fits within the extreme sport category. The lack of consensus on the definition of extreme sport has resulted in a range of different terms being used interchangeably [e.g., (8, 9, 11, 13)].

Lastly, the systematic review was limited to using studies with participants over the age of 18. This removed some potentially informative papers that may have added insight into the motives of extreme sport participants [e.g., (65, 94–96)]. This age limit was put in place to ensure participants within studies had greater experience and emotional maturity. However, given the early adoption/engagement of some sports linked to extreme sport [e.g., skiing: (97)], it is possible that many adolescents might have sufficient experience, and that early adoption might contribute to some of our themes (e.g., risk management). In addition, the age limits used here meant we did not consider how motivation could change as an athlete gets older and progresses through their lifespan. It is reasonable to assume that athletes motives and risk perception may change as they age and that participation may continue into old age. The British Mountaineering Council (BMC) conducted a review of membership survey in 2017 and found participants ages ranged from 18 to beyond 65, highlighting the wide age range of participation in these sports. However, like our study the survey did not include individuals under the age of 18 (98).

## Conclusion

This review demonstrates that there are multiple different reasons individuals are motivated to participate in extreme sport and that researchers need to consider the subjective nature of the different motives. There are many reasons for this, but the predominant one that is clear in the current results is that different perceptions of risk can change an individual's response to motivation.

Our results also highlight the complexity of understanding extreme sport in more depth and therefore the future research needed to unpack this area in more detail. We hope this review will encourage researchers to continue researching extreme sports to understand more globally the reasons for individual participation and how these link to other “risky” situations.

## Data Availability

The datasets presented in this study can be found in online repositories. The names of the repository/repositories and accession number(s) can be found in the article/Supplementary Material.

## Footnotes

^1^Statcheck is an online tool used to screen for errors in the reporting of *p* values from frequentist statistical tests (23).
